# A novel technology of solarization and phytoremediation enhanced with biosurfactant for the sustainable treatment of PAH-contaminated soil

**DOI:** 10.1007/s10653-022-01460-0

**Published:** 2023-01-03

**Authors:** Anthony Esimajemite Futughe, Huw Jones, Diane Purchase

**Affiliations:** 1 Eco-Remediation Technologies & Consultants Ltd., Beech Hill Court, 240-242 Dunstable Road, Luton, LU4 8JL UK; 2 Department of Natural Science, Faculty of Science and Technology, Middlesex University, The Burroughs, London, NW4 4BT UK; 3 Advanced Bacterial Sciences Limited, Third Floor Crown House, 151 High Road, Loughton Essex, IG10 4LG, UK

**Keywords:** Soil solarization, Phytoremediation, Contaminated soil, Biosurfactant, Polycyclic aromatic hydrocarbons (PAHs), Niger delta

## Abstract

Hydrocarbon-contaminated land has been a significant issue throughout Nigeria’s Niger Delta since the discovery of crude oil in 1956. This paper proposes a novel and sustainable technique involving soil solarization, phytoremediation and biosurfactant to treat polycyclic aromatic hydrocarbon (PAH) contamination. The treatment effect on PAH reduction, plant growth, rhizosphere microorganisms and their enzymatic activities was evaluated. Twenty-eight days of solarization was carried out before the introduction of *Chromolaena odorata* seedlings for an 84-day phytoremediation period using a 4 × 4 (vegetated) and 2 × 4 (non-vegetated) cell microcosms to simulate the Niger Delta’s subtropical conditions. Soil solarization resulted in significant PAH reduction (*p* ≤ 0.01) of phenanthrene, fluoranthene and benzo(a)pyrene with means reduction of 60.0%, 38.7% and 36.1% compared to their non-solarized counterparts with 18.0%, 18.0% and 18.8% at 95% CI (32.7, 51.3), (15.4, 26.1) and (8.0, 26.6), respectively. In post-solarization, all solarized and vegetated treatment groups significantly reduced (*p* ≤ 0.01) PAHs compared to their respective counterparts, while biosurfactant contribution in this combination was negligible (*p* ≥ 0.05). The growth parameters of *C. odorata,* total soil/rhizosphere heterotrophic microorganisms and their enzymatic activities of dehydrogenase and urease increased in all solarized treatments indicating essential biochemical processes. The novel and successful integration of soil solarization and phytoremediation using indigenous *C. odorata* as a combined technique to treat even the most recalcitrant form of hydrocarbons (PAHs) brings up new opportunities for a sustainable method of cleaning up contaminated land in Nigeria's oil-rich Niger Delta.

**Graphical Abstract:**

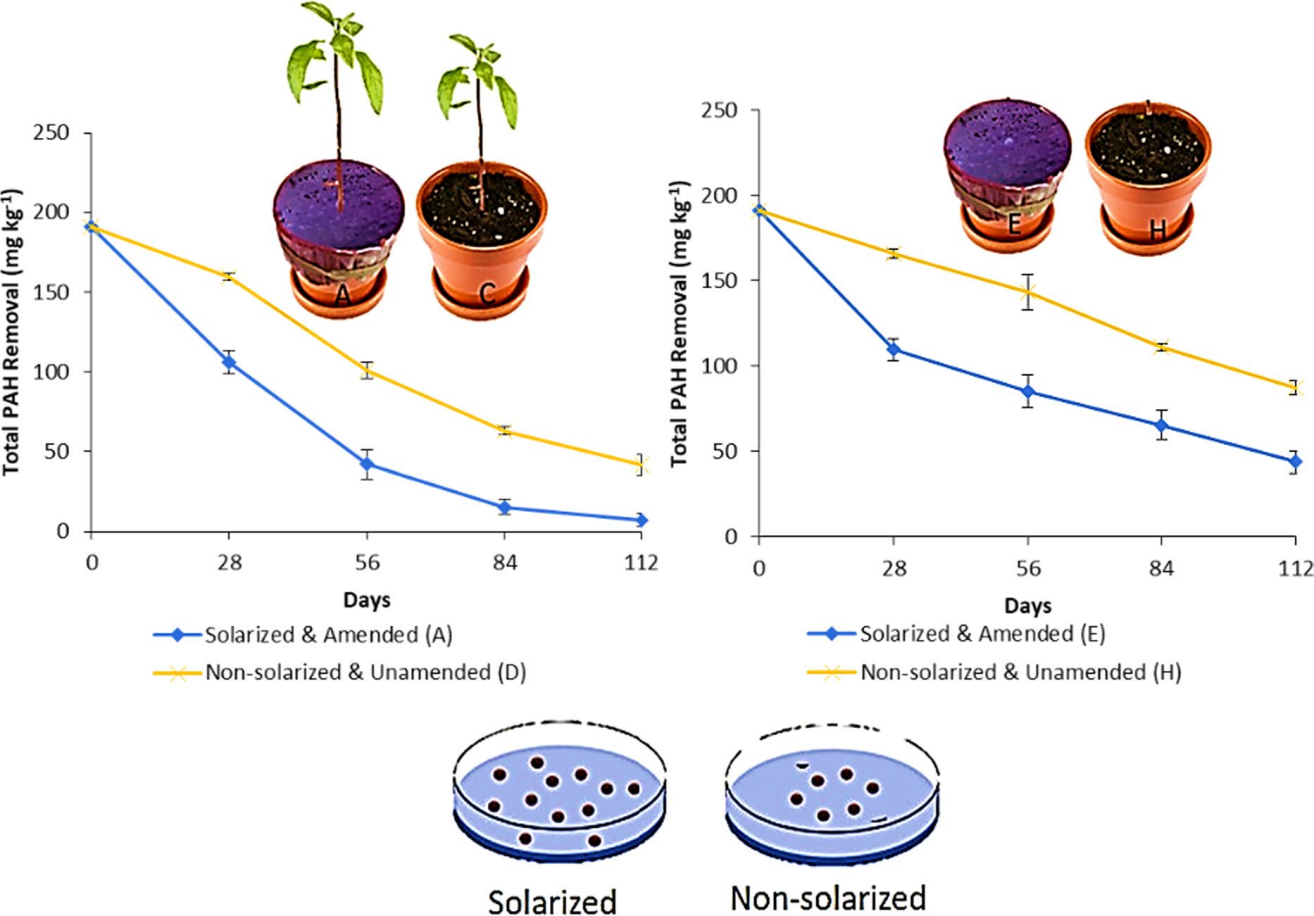

**Supplementary Information:**

The online version contains supplementary material available at 10.1007/s10653-022-01460-0.

## Introduction

The Niger Delta region, Nigeria is an area that exemplifies a strong, long-standing connection between people and the environment. It is the world’s second-largest delta, third-largest wetland, and largest wetland in Africa (Anifowose, 2008; Chinweze and Abiola-Oloke, 2009; Fatoyinbo & Simard, 2013). It is among the ten most important wetlands and marine environments on the earth (FME et al., 2006; ANEEJ, 2004). As the largest natural gas deposit and second-largest oil reserve on the continent, the region is the primary oil producer. Over 20% of Nigeria's GDP, 95% of its foreign exchange earnings, and more than 65% of its budgetary income come from the crude oil industry (FOE, 2004). According to the Energy Information Administration (EIA), Nigeria produced 1.2 trillion cubic feet of dry gas in 2012, placing it 25th in the world (Okoye et al., 2022). Nigeria's oil exports are worth $89 billion annually (Könnet, 2014; OPEC, 2015) with more than $600 billion in revenue generated from crude oil production since 1960 (Ite et al., 2013). However, a variety of anthropogenic activities, such as oil and gas extraction, are putting the Niger Delta's rich biodiversity under threat. Considerable volumes of crude oil and its processed products are accidentally spilt into the region’s natural environment by refineries, oil tankers, and offshore vessels. Oil pipelines are also vandalized and corroded as they age. Flow station oil blowouts, oil sabotage, and theft combined with illegal bunkering are other ways that oil is released into the environment. There are over 17,000 compounds of complex hydrocarbon mixtures in crude oil including polycyclic aromatic hydrocarbons (PAHs) which are considered persistent organic pollutants (POPs) with two or more fused benzene rings (Marshall & Rodgers, 2004; Oluseyi et al., 2011). They are created by the incomplete combustion of coal, oil, wood or other organic materials and are extremely lipophilic and pervasive in the environment (Sun et al., 2009; Wang et al., 2012). PAHs have a propensity for bioaccumulation and lead to adverse health impacts as some are highly carcinogenic or mutagenic (IARC, 1983).

The Sustainable Development Agenda, which includes sustainable consumption and production practices in Sustainable Development Goals (SDG) 12, considers the issue of soil contamination on a worldwide scale (WHO, 2021). Conventional remediation approaches often consume energy and water, produce wastes, lead to atmospheric pollution and generate greenhouse gas (Futughe, 2012; Schröder et al., 2008). Sustainable remediation has become the focus of much international research. Sustainable remediation, according to SuRF-UK (2009) is *“the practice of demonstrating, in terms of environmental, economic and social indicators, that the benefit of undertaking remediation is greater than its impact, and that the optimum remediation solution is selected through the use of a balanced decision-making process”* and by USSuRF (2009) as *“a remedy or combination of remedies whose net benefit on human health and the environment is maximized through the judicious use of limited resources”.*

Due to financial limitations, ex situ treatment cannot be used on the vast amount of contaminated land in the Niger Delta region; as a result, cost-effective sustainable remediation technologies, such as solarization and phytoremediation supplemented with biosurfactant, are needed. Phytoremediation is applicable to both organic and inorganic contaminants and is considered a sustainable remediation option with the added advantage of utilizing microorganisms to increase the rate of remediation (Futughe et al., 2020; Gabriele et al., 2021). The environmental friendliness, biodegradability, reduced toxicity and cost-effectiveness of biosurfactants, make them sustainable compared to their chemically synthesized counterparts (Rahman et al., 2003; Das and Mukherjee, 2008; Souza et al., 2014). Numerous reports have shown how biosurfactants can increase the availability of hydrocarbon pollutants and the ensuing biodegradation (Cheng et al., 2018; Liang et al., 2017; Liao et al., 2015; Shah et al., 2016). Soil solarization is beneficial to environmental sustainability as it leaves no toxic residues in the environment while inducing complex physical, chemical and biological changes which include soil structure improvement, increase in mineral nutrient availability and soluble organic matter that impacts positively on plant growth and yields, microflora and microfauna population in the soil with high influence on enzyme systems (Chen et al., 2000; Emoghene & Futughe, 2011; Gamliel & Katan, 2012; Al-Shammary et al., 2016; Díaz-López et al., 2021). Although enhanced rhizoremediation with solarization has been investigated on crude oil-contaminated swamp soil with over 67% total petroleum hydrocarbon reduction (Ubogu et al., 2019), the application of soil solarization combined with phytoremediation to treat contaminated land remains under-researched. The integration of soil solarization and phytoremediation enhanced with biosurfactant in the currently presented work is the first of its kind. Although biosurfactants have been reported to enhance phytoremediation (Futughe et al., 2020; Liao et al., 2016; Posada-Baquero et al., 2020), their combined application with soil solarization has never been carried out anywhere in the world. In order to treat PAH-contaminated soil modified with biosurfactant, this study evaluated the possibility of soil solarization combined with phytoremediation. Using laboratory microcosms designed to mimic the contaminated land conditions in the Niger Delta, this study assessed the impact of solarization on PAH reduction, plant growth, rhizosphere microorganisms and their enzymatic activity.

## Materials and methods

### Reagents and chemicals

All chemicals and reagents used in this research were obtained from Fisher Scientific and Sigma-Aldrich (UK) and were of analytical grade quality or above. Phenanthrene (C_14_H_10_), fluoranthene (C_16_H_10_) and benzo[a]pyrene (C_20_H_12_) were chromatography grade while dichloromethane (DCM) (CH_2_CL_2_), acetone (C_3_H_6_O), hexane (C_6_H_14_) and ethanol (C_2_H_6_O) were high-performance liquid chromatography (HPLC) grade.

### Biosurfactant analysis

A commercially available rhamnolipid (R90 Rhamnolipid biosurfactant) with a critical micelle concentration (CMC) and half maximal effect concentration (EC_50_) of 105 and 0.1 g l^−1^, respectively, produced by separation and purification processes using *Pseudomonas aeruginosa* in Canola oil substrate was purchased from AGAE Technologies, USA.

### Soil sampling

A dark yellowish brown arable soil type previously characterized by Kay (1936) and Revitt et al. (2014) was collected from the surface to a depth of 25 cm at Sonning Farm (University of Reading, Berkshire, UK), which is situated on an alluvial plain of the River Thames with GPS coordinates N51′28.898 W00′53.844. The soil has a sandy loam texture and characterized as Chromic Endoskeletic Luvisols. Samples were transported to a laboratory where it was thoroughly homogenized by mixing and air-dried at room temperature (28 ± 2 °C) for 6 days to retain the viable microorganisms before being passed through a 2 mm sieve.

### Soil physico-chemical parameters

The following physico-chemical properties were determined using conventional techniques: the ‘key for finger assessment of soil texture guideline’ by Thien (1979); soil pH using pH meter with combined electrode; soil moisture content related to an air-dried basis by Hesse (1971); soil organic matter content using loss by ignition method procedure according to Schulte and Hopkins (1996); soil nitrate (NH_3_^−^-N) extraction; available P was determined using a modified procedures of a single-solution reagent for colour development in the soil extract that contains ammonium molybdate, ascorbic acid, and a trace amount of antimony (Murphy and Riley (1962), Watanabe and Olsen (1965), Olsen and Sommers (1982)); the Chapman (1965) technique was followed to determine the soil cation exchange capacity (CEC) using the sodium acetate method; soil background heavy metals (Cd, Cr, Cu, Fe, Mn, Ni, Pb and Zn) and 16 EPA PAHs were carried out using Inductive Coupled Plasma Optical Emission Spectrometry (ICP-OES) (Thermo Scientific iCAP 6000 series) and modified ultrasonic extraction methods of Fan et al. (2008) and Song et al. (2006), respectively (see supplementary material).

### Experimental design

*Chromolaena odorata*, a plant typically found in Nigeria, was purposefully selected for this study from a contaminated site in River State, Nigeria, in Bomu Manifold, K-Dere, Gokana Local Government Area (Ogoniland) (GPS coordinates of 4°39′44.6’’N 7°16′40.1’’E). Its selection as the experimental plant was based on its local abundance, ease of cultivation and sustainability advantages of the use of indigenous flora. Air-dried soil from Sonning Farm was contaminated artificially with a blend of benzo[a]pyrene, fluoranthene, and phenanthrene (PAHs). A spiking protocol as demonstrated by Jacobsen et al. (2002) was employed. A 25% fraction (250 g) of the soil sample was spiked with 240 mg (80 mg each) of PAHs, which were then dissolved in 25 ml of acetone. The flask was then closed for 5 min to allow the solvent to disperse. Overnight, the solvent evaporated and the 75% (750 g) of the soil subsample that was left was mixed thoroughly and amended with 16.7% (dry weight) of air-dried screened (< 2 mm) commercially prepared compost. This gave a solvent concentration of 10% (v w^−1^) in the treated fraction of the soil sample. To ensure an even distribution of the soil-PAHs-compost amendment, the amended compost soil mixtures were well mixed and passed three times through a 2-mm steel gauge sieve. The soil-PAHs-compost amendment was randomly sampled in duplicate to test for homogeneity and a satisfactory result showed 73.35 ± 7.11, 74.94 ± 10.39 and 78.44 ± 0.93 mg kg^−1^ (mean ± SD) for spiked 80 mg kg^−1^ each of phenanthrene, fluoranthene and benzo[a]pyrene, respectively, as a baseline from literature (Ayodele et al., 2015). The experimental design as shown in Table 1 incorporated treatment with or without the rhamnolipid biosurfactant (500 mg kg^−1^). Control soil was treated with acetone only.

**Table 1:** Experimental design of treatment groups

Sample	Vegetated treatment *C. odorata-*(240 mg kg^−1^ PAHs) (*n* = 4)			
	Solarized and amended	Solarized and unamended	Non-solarized and amended	Non-solarized and unamended
A	+	−	−	−
B	−	+	−	−
C	−	−	+	−
D	−	−	−	+
*Unvegetated control (240 mg kg*^*−1*^* PAHs) (n = 2)*				
E	+	−	−	−
F	−	+	−	−
G	−	−	+	−
H	−	−	−	+

+  = Presence of treatment. − = Absence of treatment

**Fig. 1:**
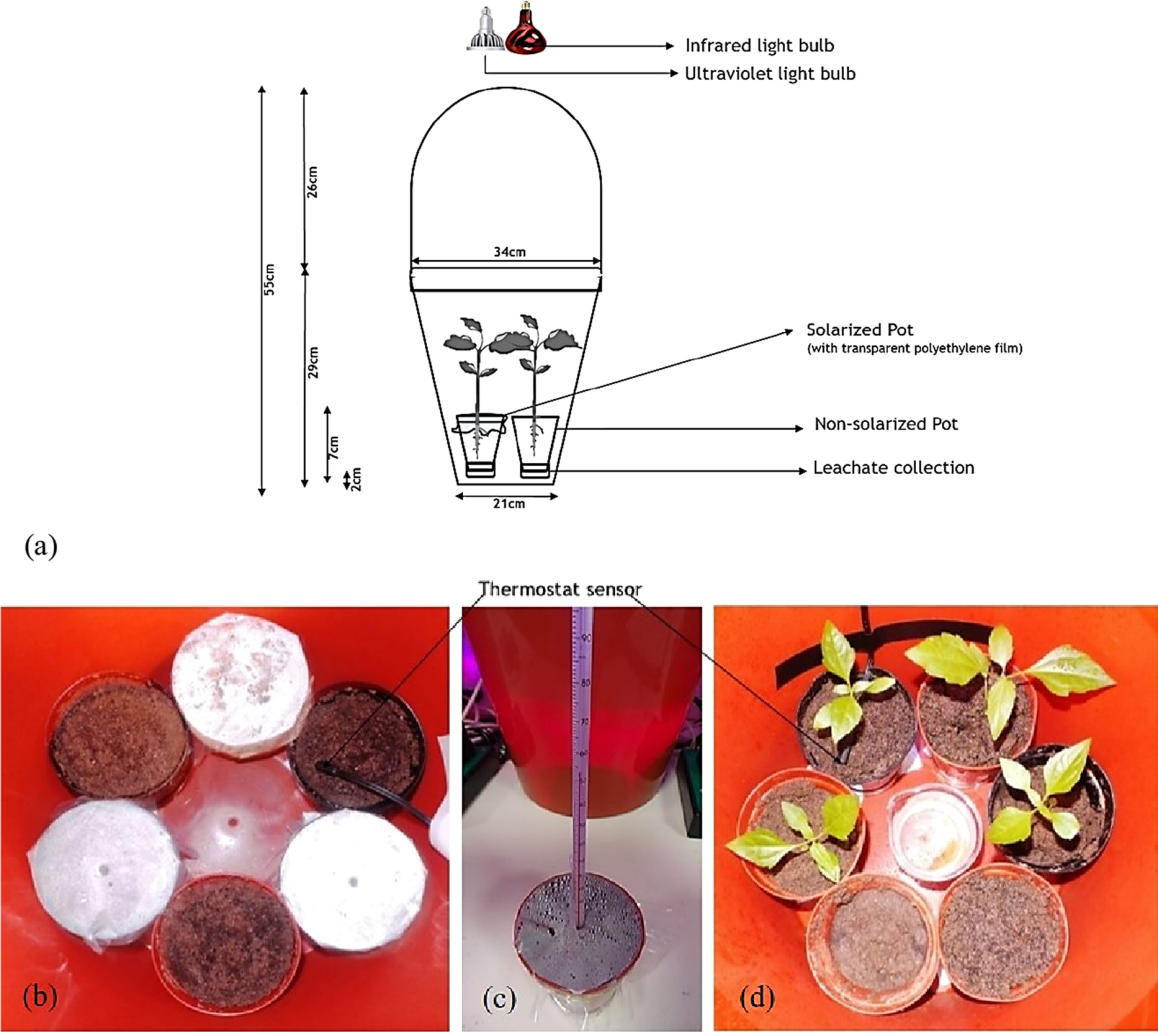
**a** Laboratory microcosms simulating the subtropical conditions in the Niger Delta region, Nigeria. **b** Treatment pots covered with transparent polyethylene sheets during 28-day solarization. **c** Piercing soil with mercury in glass thermometer for soil temperature readings at 1 cm dept. **d** Transplanting of seedlings of the same aged after solarization. With vegetative and unvegetative treatments consisting of randomly arranged 4 × 4 and 2 × 4 cells microcosm design, respectively

A microcosm was designed to accommodate plant growth and to allow soil and leachate collection. Infrared (50 W) and LED (visible) bulbs (5 W) were used to simulate incoming solar radiation as 90% of solar radiation is of these wavelengths (Zhu et al., 2003). The light was controlled automatically to simulate day and night and the temperature was regulated by Biogreen Digital Thermostat. The daylight simulations were set for 10 h while the non-solar radiation period was 14 h (night) as shown in Fig. 1 to replicate a comparable sunshine (heat level) in the region. Watering was undertaken to field capacity with artificial rainwater (0.01 M of CaCl_2_). Soil solarization was carried out with transparent polyethylene films for 28 days before transplanting *C. odorata* seedlings of the same age for a further 84-day phytoremediation period. Soil temperatures were measured at 1 and 4 cm depths, respectively, by the insertion of mercury in glass thermometers. Vegetated and their unvegetated counterparts consisted of randomly arranged 4 × 4 and 4 × 2 cell microcosm designs, respectively (Fig. 1a–b).

### PAHs extraction and analysis

Extraction of PAHs from soil samples was achieved using the method of Fan et al. (2008). Air-dried soil (5 g) was weighed, combined with 25 ml of dichloromethane (DCM), and then extracted three times for 1 h each using an ultrasonic bath (Clifton sw30H), with the water kept at 35 °C. The combination was centrifuged (Eppendorf 5702, UK) at 4000 rpm for 5 min to separate the supernatant from the soil, and it was then filtered into 20 ml vials and kept in the refrigerator at 4 °C in preparation for clean-up and analysis. Solid phase extraction (SPE) clean-up was carried out with a 12-port vacuum manifold from SUPELCO with 1 g per 6 ml ENVI™-Florisil glass cartridges. After conditioning the sorbent of the SPE cartridges, 3 ml of the supernatant was filtered through the column and was consecutively eluted with 6 ml hexane and DCM mixture of 1:1. For GC-FID analysis, the mixed eluate was reconstituted in hexane with a final volume of 2 ml after being fully dried under a mild nitrogen stream. Samples extracts (1 µl) were analysed by a Shimadzu GCMS–QP 2010 and a DB-5 capillary column (30 mm × 0.25 mm × 0.25 µm). The following program was used to achieve separation: the oven's temperature was initially set at 80 °C (held for 1 min), then increased to 275 °C at 15 °C min^−1^, held for 1 min; 285 °C at 10 °C min^−1^, maintained for 1 min; and finally elevated to 295 °C at 5 °C min^−1^, held for 1 min. Both the carrier gas (1.5 ml min^−1^) and the make-up gas (35 ml min^−1^) used were helium. The extract was injected in the splitless mode using a 1.0 µl aliquot. The detector was kept at 300 °C, while the injector was kept at 250 °C.

By dividing the difference between the current PAH readings and the starting PAH value, as shown in the following equation, the percentage of PAH degradation on each sampling day was calculated:

$$PAH\%\, removal = \frac{{PAH_{0} - PAH_{SD} }}{{PAH_{0} }}$$

where PAH_0_ = total polycyclic aromatic hydrocarbon on day 0 of the sampling and.

PAH_SD_ = total polycyclic aromatic hydrocarbon on each sampling day.

### Quality control and quality assurance

All chemical extractions were done with two blank samples per analysis, and a minimum of one blank per set of samples was extracted. Samples had four replicates except when otherwise stated. Standard aseptic technique was strictly followed, and experiments on PAHs recovery were carried out by spiking a known concentration (1 mg kg^−1^) of phenanthrene, fluoranthene and benzo[a]pyrene standards to uncontaminated soil. The results showed satisfactory recovery of greater than 90, 80 and 70%, respectively, for phenanthrene, fluoranthene and benzo[a]pyrene with a detection limit of 0.001 mg g^−1^ of soil.

### Enumeration of soil/rhizosphere total heterotrophic microorganisms

Serial dilution and pour plate techniques were used to enumerate rhizosphere microorganisms. Aqueous suspension of a 1 g soil sample from the rhizosphere had its microbial population serially diluted. Pour plates of each of the serial dilutions were prepared using approximately 20 ml of molten. Glycerol Yeast Extract Agar (GYEA) for actinomycetes, Sabouraud Dextrose Agar (SDA) for fungi, and Tryptic Soya Agar (TSA) for bacteria were evenly mixed by swirling and allowed to set (solidify). Three replicate pour plates and their controls for each dilution were inverted and incubated at 25 °C for 3 to 7 days for the isolation of bacteria, actinomycetes and fungi. After being incubated at 25 °C for 3 to 7 days, distinct bacterial, actinomycetes and fungal colonies developed on each Petri dish. These colonies were enumerated, and the colony-forming units per gram (cfu g^−1^) were calculated.

### Soil enzymatic activity

#### Dehydrogenase activity

Using the Guan (1986) designed technique, the reduction rate of 2,3,5-triphenyltetrazolium chloride (TTC) to a red water-insoluble triphenylformazan (TPF) was determined. A 3 g soil sample, 0.03 g of CaCO_3_ and 0.5 ml of 3% tetrazolium chloride (TTC) were combined in a shaker before being incubated at 37 °C for 24 h in the dark. The mixture was extracted for 1 min with a 5 ml addition of methanol. Glass funnels pre-packed with absorbent cotton at the bottom were used to filter the solution into a 50 ml volumetric flask. Methanol was used to wash the tube until the red colour disappeared on the absorbent cotton in the funnels. After being diluted to 50 ml using a spectrophotometer, the samples were measured at 485 nm. A control assay was carried out simultaneously without CaCO_3_ and TTC. Soil dehydrogenase activity was determined by extrapolated values obtained against a standard calibration curve of TF and reported as the µg TPF g^−1^ dry soil/24 h.

#### Urease activity

Urease activity was measured using the approach of Guan (1986) and Yang et al*.* (2006). Briefly, a mixture of 5 g of air-dried soil sample and 1 ml of toluene was left for 15 min before adding 10 ml of 10% urea followed by 20 ml of pH 6.7 citrate buffer that was thoroughly mixed and then incubated for 24 h at 37 °C. Following incubation, samples were completely diluted with distilled water at 37 °C and oscillated before being promptly filtered. A 50 ml volumetric flask containing 3 ml of the filtrate was then filled with 10 ml of distilled water, 4 ml of sodium phenate (1.35 M), and 3 ml of sodium hypochlorite (active chlorine 0.9%). After being left for 20 min, the flask was diluted to volume. By using a reference-calibrated curve generated by the Indophenol Blue Method at 578 nm, the concentration of NH_4 _^+^ ions generated by urea hydrolysis was quantified calorimetrically as the blue coloured complex of urease activity. Each sample was made using a urea-free control. The amount of NH_4_^+^-N produced by 1.0 g of air-dried soil at a rate of 37 °C h^−1^ was used to define a unit of urease activity.

#### Plant analysis

The plants were taken after 84 days of growth, and their height was measured after taking into account the growth differences from transplants. The length of the roots from the stem base to the longest root tip of the plant was also measured. With the soil still firmly adhered to the plants' fibrous roots, shoots and roots were removed from pots. To remove soil particles, they were then washed with deionised water and dried with tissue paper. According to Campbell and Plank (1998), the plant material was dried in an oven at 70 °C overnight and the dry weights recorded.

#### Statistical analysis

The experimental results were statistically analysed using Minitab®18 statistical software and all results were deemed significant at a 95% confidence level (*p* ≤ 0.05). All treatments had four and two replicates for vegetated and unvegetated groups, respectively, except where it was otherwise stated and are reported as mean ± standard deviation (SD). Differences between samples were analysed with either two-sample t-tests or analysis of variance (ANOVA) with post hoc analyses using Tukey pairwise comparisons with Bonferroni correction for Type 1 error inflation. Homogeneity of variance and normality assumptions were checked using Bonnet’s variances test and Anderson–Darling normality tests, respectively. Relationships between dependent variables (% PAHs removal efficiency, plant growth parameters, total heterogeneous microorganisms and soil enzymatic activity) and the treatment independent variables (solarization, biosurfactant and/or vegetation) were investigated using the general linear model (GLM) procedure.

## Result and discussion

### Soil physico-chemical parameters and solarization effect on soil temperatures

The soil analysis using standard methods gave the following data: clay loam; moisture content 13.7%; organic matter content 2.48%; pH 7.14; Nitrate (NO_3_^−^-N) extraction 18 mg L^−1^; available P 0.32 mg g^−1^; CEC 17.8 meg 100^–1^ g; heavy metals < 0.1 mg g^−1^ and initial PAHs were non-detectable. These results are transferable considering the Niger Delta's wide range of soil textures (Kamalu et al., 2002).

**Fig. 2:**
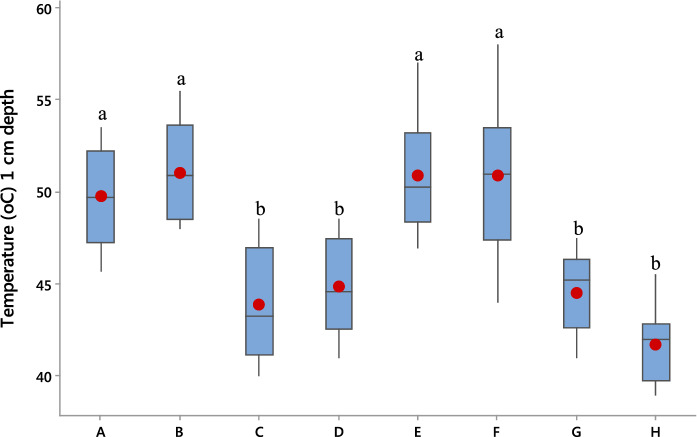
Box plots of pre-vegetated and unvegetated treatments showing soil temperatures for solarized and non-solarized soils with or without biosurfactant-amendment at 1 cm depth. Means with different letters are significantly different (*p* ≤ 0.01). A = Solarized & amended (Pre-vegetated). B = Solarized & unamended (Pre-vegetated). C = Non-solarized & amended (Pre-vegetated). D = Non-solarized & unamended (Pre-vegetated). E = Solarized & amended (Unvegetated). F = Solarized & unamended (Unvegetated). G = Non-solarized & amended (Unvegetated). H = Non-solarized & unamended (Unvegetated)

The soil temperature results obtained with the microcosm during soil solarization by covering it with or without a transparent polyethylene sheet indicated successful simulations especially with solarized treatment at both 1 and 4 cm depths, respectively. The reported temperature range of the highest mean for solarized treatment with 51.0 and 48.3 °C compared to the non-solarized counterpart with 44.3 and 42.0 °C at 1 and 4 cm depths, respectively, agrees with previous reports (Fig. 2) (Pinkerton et al., 2000; Emoghene & Futughe, 2011; Özyılmaz, 2019; Al-Shammary, 2020).

### Effect of solarization on PAH reduction

**Fig. 3:**
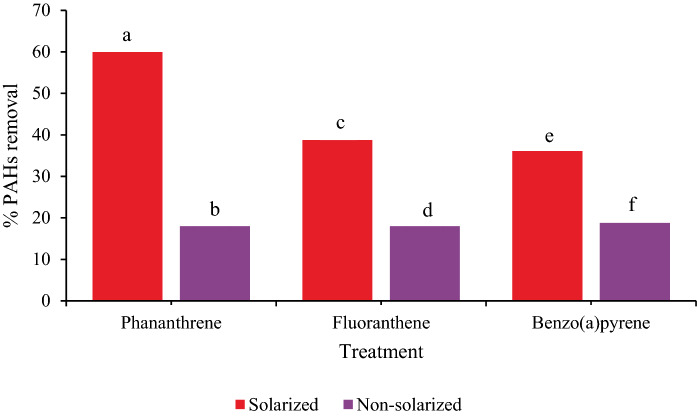
28 days soil solarization impact on % removal of phenanthrene, fluoranthene and benzo[a]pyrene prior to planting (with and without biosurfactant-amendment). Means with different letters are significantly different (*p* ≤ 0.01)

**Fig. 4:**
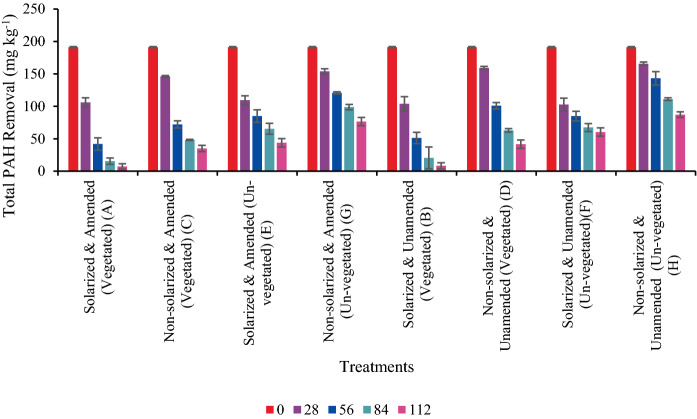
Mean reduction of total PAH removal with or without biosurfactant-amendment in solarized vs non-solarized vegetated and/or unvegetated treatments, respectively. Error bars indicate mean ± SD of four and two sampled pots for vegetated and unvegetated treatments, respectively

The effect of soil solarization on the PAH mixtures was significant (*p* ≤ 0.01) in their % removal after the 28-day solarization period. Phenanthrene with a mean reduction of 60.0% compared to its non-solarized counterpart with 18.0% has an estimated difference of 42.0% between solarized and non-solarized treatments at 95% CI (32.7, 51.3), followed by fluoranthene with a mean reduction of 38.7% compared to its non-solarized counterpart with 18.0% with 21.0% estimated difference between them at 95% CI (15.39, 26.11) and benzo(a)pyrene with a mean reduction of 36.1% compared to its non-solarized counterpart with 18.8% with 17.0% estimated difference between them at 95% CI (8.02, 26.62) as shown in Fig. 3. The effect of post-solarization was observed in total PAH reduction with statistical significance (*p* ≤ 0.01) of 41.9 mg kg^−1^, 15.4 mg kg^−1^ and 7.28 mg kg^−1^ in solarized and biosurfactant-amended vegetated treatment (A) compared to reductions of 72.1 mg kg^−1^, 48.2 mg kg^−1^ and 35.0 mg kg^−1^ in non-solarized and biosurfactant-amended vegetated counterpart (C) at days 56, 84 and 112, respectively, as shown in Fig. 4 (page 24). There was also a relatively significant reduction (*p* ≤ 0.01) in total PAHs in the unvegetated treatment groups with or without biosurfactant-amendment with 84.9 mg kg^−1^, 65.4 mg kg^−1^and 43.9 mg kg^−1^in solarized and biosurfactant-amended unvegetated treatment (E) compared to reductions of 120.8 mg kg^−1^, 98.8 mg kg^−1^ and 76.5 mg kg^−1^in non-solarized and biosurfactant-amended unvegetated counterpart (G) at days 56, 84 and 112, respectively (Fig. 4). There was a statistical significance in the reduction of total PAH mixtures with *p*-value, *t*- and *F*- statistics of 0.00, 5.08 and 25.9 with R-square (adjusted) of 88.4% using a general linear model between solarized and vegetated/unvegetated treatments (A, B, E and F) and non-solarized and vegetated/unvegetated treatments (C, D, G and H) with or without biosurfactant-amendment. The linear model shows that total PAH will be reduced by an additional further average of 8.6% in the presence of soil solarization compared to non-solarized treatments, under various conditions of vegetation and/or biosurfactant presence over 112 days (Table 2). According to this study's findings, the daily simulated temperatures of solarized moist soil treatments gradually increased. This may have affected the soils' physical, chemical, and biological characteristics, such as increasing the amount of soluble organic matter and readily available minerals (e.g. N mineralization, Ca, Mg, P, and K). This was accomplished by enabling fast decomposition of organic matter utilizing the heat produced beneath the transparent polyethylene sheet. This direct impact from solarization creates a favourable microenvironment for bacterial metabolic activity and ultimately, PAH biodegradation. According to Leahy and Colwell (1990); Zhang et al. (2005); and Okere and Seme (2012) increase in temperature up to an optimum of 30 to 40 °C results in a corresponding increase in bacterial metabolic activity and PAH biodegradation due to high temperature adaptation by PAHs degrading bacteria while maintaining their metabolic activity. Other studies have shown that an increase in the soil temperature could result in increasing solubility. According to Ghosal et al. (2016), Margesin and Schinner (2001) an increase in temperature leads to an increase in PAHs solubility which in turn increases the bioavailability of PAH molecules. Thus, the significant removal of PAHs from solarized soils may be attributed to the physico-chemical and/or biological processes as both are enhanced by increased soil temperatures. According to studies by Miller et al. (1989) and Podoll et al. (1989), increasing soil temperature decreases PAHs sorption by soils, increases their solubility and vapour pressure, and significantly speeds up PAH biodegradation in polluted areas (Ghosal et al., 2016) as sorption and volatilization are the primary mechanisms for abiotic elimination of PAHs from soil (Bulman et al., 1985; Park et al., 1990).

**Table 2:** Summary of coefficients and associated statistical parameters for general linear model analysis of treatment response variables and experimental conditions

Treatment	Factors	Coef	SE Coef	T-Value	F-Value	P-Value	R-Square (adj.)%
% PAH removal	Solarization (S & NS)	-8.56	1.68	−5.08	25.85	0.00	88.39
	Biosurfactant (B & NB)	1.79	1.68	1.06	1.13	0.30	
	Plant (V & UV)	−7.29	1.68	−4.33	18.75	0.00	
	Time	0.98	0.043	15.98	255.30	0.00	
Rhizosphere microorganisms (CFU g^−1^ × 10^4^)	Solarization (S & NS)	−14.80	9.22	−1.61	2.58	0.12	63.98
	Biosurfactant (B & NB)	2.00	9.22	0.22	0.05	0.83	
	Plant (V & UV)	−47.17	9.22	−5.12	26.17	0.00	
	Time	1.55	0.23	6.67	44.48	0.00	
Soil Enzymatic activity (μg g^−1^ dry soil)	Solarization (S & NS)	−1.63	0.93	−1.76	3.10	0.09	34.46
	Biosurfactant (B & NB)	0.81	0.93	0.88	0.77	0.39	
	Plant (V & UV)	−2.61	0.93	−2.82	7.95	0.01	
	Time	0.08	0.02	3.56	12.69	0.00	

*S* Solarized, *B* Biosurfactant amended, *V *Vegetated, *NS* Non-solarized, *NB* No Biosurfactant amended, *UV* Unvegetated

According to Miller et al. (1989), the majority of soil heterotrophic bacteria are mesophiles, which have a growth range of 10–15 °C to 45 °C and an ideal temperature of about 25–35 °C. However, a drop in temperature slows down the rate of biochemical reactions as well as the growth and development of these communities of heterotrophic microorganisms. PAHs biodegradation has also been reported to take place over a wide range of temperatures, Lau et al. (2003) reported optimum temperatures of > 50 °C and > 75 °C in the degradation of PAHs in spent-mushroom compost. They reported that over 90% of PAHs removal took place at these very high temperatures. Similarly, PAHs biodegradation has been reported at very high temperatures (60–70 °C) by *Thermus* and *Bacillus* spp. (Feitkenhauer et al., 2003). Studies have shown that microorganisms have adapted to metabolize PAHs at extreme temperatures; however, most reports focus on mesophilic temperature instead of the efficiency of transformations at very high or low temperatures (Bamforth & Singleton, 2005).

The effect of post-solarization of PAH mixtures’ significant removal/degradation, on the other hand, could be based on increased desorption, total heterogeneous microbial activity, soil/rhizosphere enzymatic activity, improved agronomic performance of plants with phytoremediation potential and/or enhanced action of catalytic substances or a combination of all of the above. Despite very limited information on the effect of solarization on contaminant removal/degradation, there are several reports on organophosphorus insecticides and benzimidazole fungicides reduction in solarized soils (Gopal et al., 2000; Yarden et al., 1989). Navarro et al. (2009) also reported on triazine and phenylurea herbicides dissipation from soil solarized with polyethylene sheet. Fenoll et al. (2010) reported increased fungicide dissipation by solarization and biosolarization concerning the control treatment and suggested the dissipation was mainly due to increased soil temperatures. The accumulation and dissipation of contaminants in soil has be demonstrated to be affected by soil solarization. Pesticide persistence can be shortened by solarization depending on the nature and time of pesticide application (Avidov et al., 1985; Rubin & Benjamin, 1983; Yarden et al., 1989). Ubogu et al. (2019) reported a 67.3% reduction of TPH on crude oil-contaminated mangrove swamp soil using enhanced rhizoremediation with solarization. In addition, soil organic amendment may affect soil pollutant degradation (Flores et al., 2008). From these findings, the novelty of integrating soil solarization as a remediation technique in treating hydrocarbon (PAHs)-contaminated land has been established. The demonstrated suitability and compatibility of soil solarization and phytoremediation suggest it can be a sustainable, environmentally friendly and cost-effective treatment option for the large area of contaminated land in the Niger Delta region, Nigeria.

### Effect of biosurfactant on PAH reduction

Biosurfactant was observed to have improved the biodegradation of PAH but the extent is not as significant (*p* ≥ 0.05) as in combination with phytoremediation. However, a vast body of literature has reported the significant role biosurfactant plays in the biodegradation of PAHs from contaminated soil (Cheng et al., 2018; Futughe et al., 2020; Gao et al., 2007; Liang et al., 2017; Shah et al., 2016). Although rhamnolipid biosurfactant may be thermal stable, the relatively high temperatures recorded during the 28-day soil solarization period for both solarized and non-solarized treatments, may have contributed to biosurfactant negligible PAH reduction. According to Lamichhane et al. (2017), the temperature has a limited impact on the solubility of PAHs with the use of surfactants; anthracene and pyrene mineralization rates were 48.8% and 66.1%, respectively, at 25 °C and 18.5% and 61.5%, respectively, at 10 °C. It was also reported that the effect of rhamnolipid biosurfactant on the solubility of naphthalene, phenanthrene and pyrene increased with temperatures up to 30 °C (Li et al., 2015b). A similar study was carried out by Peng et al. (2015) to investigate rhamnolipid biosurfactant-enhanced remediation of PAHs at a temperature range of 15 to 50 °C and reported that the breakdown of PAHs occurs most efficiently at a temperature of 35 °C, with anthracene and pyrene degrading by 37.5 and 25.6%, respectively, at this temperature. However, contrary to the above findings, Peng et al. (2011) observed PAH removal performance was not affected by the use of surfactant at temperatures between 10 and 40 °C, and Zhou et al. (2019) reported that temperature had no impact on rhamnolipid biosurfactant performance throughout a wide range of temperatures, from 20 to 80 °C.

Interestingly, however, the increase in soil temperature caused by soil solarization appears to have increased PAH solubility and thus bioavailability (Fenoll et al., 2010; Ghosal et al., 2016; Margesin & Schinner, 2001). This suggests the possibility that soil solarization may have also played the role of biosurfactant in solubilizing and subsequently making PAHs bioavailable for degradation as a result of the direct impact of soil temperatures.

### Effect of solarization on plant growth parameters

The effect of treatment factors on plant growth shows that solarization significantly increased (*p* ≤ 0.01) *C. odorata’s* growth throughout the phytoremediation period. A significant combined increase in heights of *C. odorata* was also seen at the end of the phytoremediation period in solarized treatments with a mean height of 24.9 cm compared to the combined mean height of 18.4 cm from non-solarized treatment groups. The plants’ shoots and roots dry biomasses were also affected by solarization significantly (*p* ≤ 0.01) with combined means of 2.95 and 2.11 g for solarized treatments, compared to their non-solarized counterparts, with combined means of 1.91 and 1.67 g for shoots and roots, respectively.

This finding on the impact of solarization on a plant is consistent with a vast body of literature on improved plant growth, yield and quality and has been attributed to soil-borne control, soil structure improvement, increase availability of N and other vital plant nutrients in addition to the greenhouse effect (Emoghene & Futughe, 2011; Al-Shammary et al., 2016; Díaz-López et al., 2021). Although correlations between performance in agronomy and phytoremediation potential may not be fully determined, *C. odorata* drastically reduced PAH mixtures significantly (*p* ≤ 0.01) with 7.28 and 8.29 mg kg^−1^ compared to their unvegetated counterparts with 43.85 and 60.31 mg kg^−1^ with and without biosurfactant amendment, respectively (Fig. 4). The impact of soil solarization on phytoremediation directly and/or indirectly from this study is promising, as a better agronomic performance of the indigenous *C. odorata* has shown significant reduction in PAHs from weathered PAHs-contaminated soil as a way of advancing phytoremediation. According to Wiltse et al. (1998), plants that are less impacted by pollutants in soils are healthier and more resilient, and as this study has shown, they will produce stronger root systems and more above-ground growth.

### Effect of solarization on soil/rhizosphere total heterotrophic microorganisms and associated enzymatic activities

**Fig. 5:**
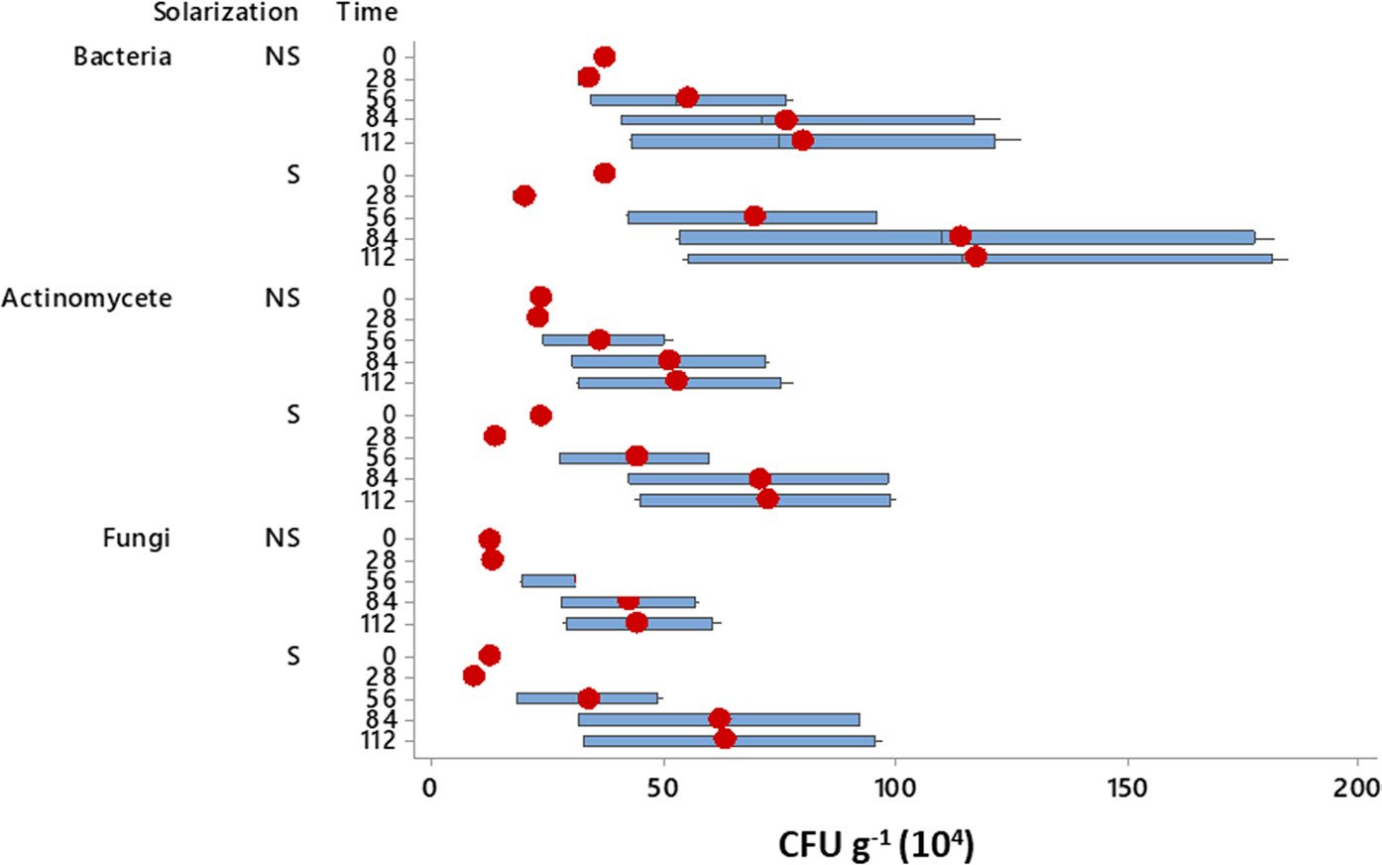
Soil solarization effects on soil/rhizosphere total heterotrophic microorganisms. *S* = Solarized. NS = Non-solarized

Solarization of the soil and its effects on total heterotrophic microorganisms shows a significant reduction (*p* ≤ 0.05) after the 28 days compared to the non-solarized treatments. However, solarization appears to have increased the density of total soil/rhizosphere heterotrophic microorganisms in all solarized treatments compared to their non-solarized counterparts but without statistical significance (*p* ≥ 0.05) at days 56, 84 and 112, respectively. In solarized treatment, bacteria had increased mean counts of 54.9, 76.6, and 79.9 cfu g^−1^ dry soil; actinomycetes had increased mean counts of 44.0, 70.8, and 72.3 cfu g^−1^ dry soil; and fungi had increased mean densities of 33.8, 62.0, and 63.4 × 104 cfu g^−1^ dry soil. Compared to mean counts of 54.9, 76.6, and 79.9 cfu g^−1^ dry soil for bacteria; 36.1, 51.0, and 53.0 cfu g^−1^ dry soil for actinomycetes; and 33.5, 42.4, and 44.3 × 104 cfu g^−1^ dry soil for fungi in non-solarized treatment at days 56, 84, and 112, respectively (Fig. 5). Reports have shown that a broad range of soil microbes in addition to major plant pathogens have been negatively impacted by soil solarization excess generated heat (Culman et al., 2006; Gelsomino et al., 2006; Palese et al., 2004; Schoenfeld et al., 2003). According to some studies (Mahmoud, 1996; Patel & Patel, 1997; Itoh et al., 2000; Barbour et al., 2002; Sharma et al., 2002), soil solarization generally results in a decrease in the total bacterial population of the soil, whereas other studies recorded a decrease in the fungal population of the soil without any effects on the bacterial population (Coates-Beckford et al., 1997; Shukla et al., 2000). However, several studies (Kaewruang et al., 1989a; Khair & Bakir, 1995; Khaleeque et al., 1999; Di Mola et al., 2021; Dáz-López et al., 2021) revealed higher total bacterial and actinomycetes population on solarized soil. This study showed that the recolonization of the rhizosphere by advantageous microorganisms shortly after the completion of a solarization treatment was the cause of the rise in total heterotrophic rhizosphere microorganisms in solarized treatments as observed by Chen et al. (1991) and Di Mola et al. (2021).

Solarization also seems to have increased the dehydrogenase enzymatic activity in solarized treatment compared to the non-solarized counterpart, but the increase was not statistically significant (*p* ≥ 0.05). There was no significant reduction (*p* ≥ 0.05) in soil dehydrogenase activity after 28 days of soil solarization in solarized treatment with a mean of 1.45 compared with a mean of 2.33 µg TF g^−1^ dry soil in non-solarized treatment from a mean of 2.13 µg TF g^−1^ dry soil at day 0. Post-solarization may have increased soil enzymatic activity of dehydrogenase in solarized treatment with means of 0.91, 16.1 and 12.5 µg TF g^−1^ dry soil compared to non-solarized counterparts with means of 0.33, 6.14 and 5.82 µg TF g^−1^ dry soil at day 56, 84 and 112, respectively. Temperature and soil water content, according to Brzezinska et al. (1998), have an indirect impact on dehydrogenase activity by altering the soil's redox status. As the microbiological redox indicators in soil, these redox transformations are strongly linked to the respiration activity of soil microorganisms and can be used as a potential indicator of microbial oxidative activities (Tabatabai, 1982; Trevor, 1984). As shown in this study, the increased temperature during soil solarization initially reduced dehydrogenase activity compared to its non-solarized counterpart but gradually increases post-solarization with increasing total heterotrophic microorganisms re-colonizing the soil, especially vegetated soil suggesting a positive response. It is generally accepted that the dehydrogenase enzyme exists as an integral component of intact cells but does not accumulate extracellularly in soil, and its activity is utilized as an indicator of biological activity in soils. Dehydrogenase can also be used to identify the kind and severity of soil pollution. McCarthy et al. (1994) showed that fly ash-polluted soil had low dehydrogenase activity but had high activity in soils polluted with pulp and paper mill effluents (Pitchel & Hayes, 1990). There have been reports of increased dehydrogenase activities at low pesticide dosages and reduced dehydrogenase activities at higher pesticide levels (Baruah & Mishra, 1986). There was a solarization effect with significance (*p* ≤ 0.05) on rhizosphere enzymatic activity of urease in solarized treatment when compared to their non-solarized counterpart with 0.04, 0.1 and 0.1 µg NH_4_^+^-N g^−1^ dry soil compared to means of 0.02, 0.04 and 0.04 µg NH_4_^+^-N g^−1^ dry soil at day 56, 84 and 112, respectively. Many factors influence urease activity in soils including cropping history, soil depth, amendment and organic matter content, background heavy metals and PAHs, in addition to other environmental factors such as temperature (Tabatabai, 1982; Yang et al., 2006). The significant increase in urease activity as observed in this study agrees with a report by Das and Varma (2011) that urease activity typically increases with temperature suggesting that higher temperatures enhance the urease enzyme's activity coefficient. According to Díaz-López et al. (2021), combined ozonation and solarization had a negative impact on both enzymatic activities and soil microbial population in pesticides soil, due to the biocidal character of ozone; but several pesticides-degrading microorganisms showed a relative increase. However, the increase of soil/rhizosphere heterotrophic microbes and corresponding dehydrogenase and urease activities in this study, suggest the compatibility of solarization and phytoremediation. Higher rhizosphere enzymatic activity is a reflection of a greater functional diversity of the microbial community with the possibility of removing both inorganic and organic pollutants (Gianfreda, 2015).

## Conclusion

A microcosm successfully mimicked contaminated land conditions in the Niger Delta region and demonstrated soil solarization significantly enhanced phytoremediation with native *C. odorata* with respect to PAHs reduction. This reduction was attributed to the increase in soil temperature through soil solarization and not through the action of biosurfactant. The integration of soil solarization and phytoremediation as a novel, environmentally friendly and economically viable treatment for petroleum hydrocarbon-contaminated land also improved agronomic performance, enhanced soil fertility and quality, as well as microbial density, diversity and enzymatic activities. Given the ideal conditions of sunlight, high humidity and extensive presence of native *C odorata,* this study opens up new possibilities for environmentally friendly methods to clean contaminated land in Nigeria's oil-rich Niger Delta.

## Supplementary Information

Below is the link to the electronic supplementary material.

Supplementary file1 (DOCX 67 kb)
