# Supplementary material for: A novel technology of solarization and phytoremediation enhanced with biosurfactant for the sustainable treatment of PAH-contaminated soil
Source: Environ Geochem Health. 2023 Jan 3;45(6):3847–63. doi: 10.1007/s10653-022-01460-0 (PMC10232648; doi:10.1007/s10653-022-01460-0)
Supplement: Supplementary file 1 — Supplementary file1 (DOCX 67 kb) [file 10653_2022_1460_MOESM1_ESM.docx]

**Supplementary Material**

**Soil texture**

About 25 g of air dried soil sample with all particles less than 2 mm was used to form a ball of about 2 cm. Deionized water (DI) (18MΩm^-2^) was gradually added to the soil until it adhered to itself and not to the hand. The ‘key for finger assessment of soil texture guideline’ by Thien (1979) was followed to determine the soil texture because texture indicates the relative content of particles of various sizes, such as sand, silt and clay in the soil and the finger assessment is quicker and reliable.

**Soil pH**

This was determined using pH meter with combined electrode. Air-dried soil (10 g) was weighed (<2 mm) into a 100 ml glass beaker, then 10 ml of DI water was added and the mixture was stirred. It was allowed to stand for 30 minutes. Suspension was stirred every 10 minutes during this period. After 1 hour, the suspension was stirred and the combined electrode was placed in the suspension (about 3 cm deep) and the pH readings were recorded after being calibrated with buffer solutions at pH4, 7 and 10 respectively.

**Soil moisture contents**

All analysis in the laboratory was related to an air dried basis and therefore must consider the actual soil moisture content (Hesse, 1971). An empty crucible was put into the muffle furnace and left over night at 105^o^C. The crucible was then removed from the furnace and allowed to cool in the desiccators and weighed. Moist soil sample [25g (± 0.001)] was introduced into the crucible and the weight was recorded. The crucible and its content were placed in the muffle furnace and left over night at 105 ^o^C after which its weight was determined when cooled. The water contents of the soil samples were determined using the following formula:

Water Content, W (%) = W_2_ - W_3_ x 100%

W_3_ – W_1_

Where W_1_ = Weight of empty crucible container

W_2_ = Weight of crucible container + moist soil

W_3_ = Weight of crucible container + oven dried soil

**Soil organic matter contents**

The loss by ignition method procedure according to Schulte and Hopkins (1996) was used to carry out this analysis. It does not involve the use of any chemical, only the use of a muffle furnace. Its principle is based on comparing the weight of a sample before and after the soil is ignited. Before ignition, sample contains organic matter, but after ignition, only the mineral portion of the soil remains. An empty crucible was put into the muffle furnace and left over night at 105 ^o^C. The crucible was then removed from the furnace and allowed to cool in the desiccators and weighed. 25 g (± 0.001) air dried soil sample was introduced into the crucible and the weight was recorded. The crucible and its content were placed in the muffle furnace and left over night at 105 ^o^C after which its weight was determined when cooled. After which it was then placed into a muffle furnace at 440 ^o^C overnight and cooled in a desiccator again after removal. For this process, the soil was analysed in triplicate. The organic matter contents of soil samples were determined using the following formulae:

Organic matter content (%) = Mass of oven dried soil – Mass of ignited (burnt) soil x 100 %

Mass of oven dried soil

**Nitrate (NO_3_^-^ – N) extraction**

25 ml of DI water was added into soil bottle containing 3.5 g of air dried soil and 1 shot Nitrate Extraction Powder was added to the bottle, capped and shook for 30 sec. The soil coagulated in the bottom of the bottle leaving a clear extract. 1.0 ml of the aqueous soil extract was pipette into a sample cell and filled up with DI water to the 25 ml mark. One NitriVer 6 Powder Pillow content was added to the cell, swirl stoppered and shake continuously for 2 min after which it was allowed to settle for 2 min. Sample (25 ml) was poured into another clean sample cell and the content of 1 NitriVer 3 Reagent Powder Pillow was added, stoppered and shook for 30 sec thereafter allowed to settle for 10 min with a pink colouration. The same process was used for the blank but without the soil sample. The Hash Spectrophotometer at 500 nm was used to take the readings.

**Available phosphorus**

The available phosphorus was determined using a modified procedures of Murphy and Riley (1962); Watanabe and Olsen (1965); Olsen and Sommers (1982). In the modified method, a single solution reagent containing ammonium molybdate, ascorbic acid and a small amount of antimony was used, for colour development in the soil extract. Air-dried soil sample (5 g) was weighed into a 250 ml Erlenmeyer flask and 100 ml of 0.5 M sodium bicarbonate (NaHCO_3_) solution was added. The flask was closed with a stopper and shaken for 30 minutes on a shaker at 200 rmp. The blank comprises one flask containing all reagents but no soil. The solution was filtered through a Whatman No. 40 filter paper and 10 ml of clear filtrate was pipette into 50 ml volumetric flask and acidified with 5 N sulphuric acid (H_2_SO_4_) to pH 5.0. DI water was added to about 40 ml volumes and 8 ml of the single reagent was added and brought to 50 ml volume. Standard curve solution and blank were also determined using the above procedure without soil sample. The absorbance of the blank, standards, and samples were read after 10 minutes at 882 nm wavelength using spectrophotometer. Phosphorus concentration in the sample was read from the calibration curve.

**Cation exchange capacity (CEC) using Sodium as index ion**

Sodium acetate method was used to determine CEC according to Chapman (1965) protocol. Well ground air-dried soil sample (5 g) was weighed into 50ml centrifuge tube. 30 ml of 1M sodium acetate reagent was added and mixture was placed into ultrasonic bath for 5 min and centrifuge at about 1000 rpm for 3 min until the supernatant was clear, decanted and discarded. 30 ml of ethanol was then added to the soil residue and agitated to remove any excess sodium acetate and placed into the ultrasonic bath for 5 min and centrifuge. The resultant supernatant was decanted and discarded, and the washing was repeated once more. The soil residue was then extracted thrice with 20 ml portions of ammonium acetate using the same above method except that all the supernatants were filtered into 100 ml volumetric flask and made up to the mark. Previously calibrated flame photometer was used to determine the sodium content of the combined extract (which was diluted) and ascertained from the standard calibration curve. The concentration of sodium present in 100 g of soil was determined by multiplying the sodium concentration obtained by the appropriate dilution factor and by 20. The outcome is divided by 23.0 (Relative Atomic Mass of Na) to give the CEC of the soil.

**Background heavy metals determination**

Background heavy metals especially, Pb, Cd, Cr and Cu due to their well-established toxicity in soil sample were determined in the laboratory. Air dried soil sample (0.2 g) was accurately weighed to the nearest mg in triplicates into a pressure resistance 50 ml quartz or TFM vessel and avoiding contact with the inside of the vessel. Concentrated nitric acid (3 ml) was added with the vessel lid closed and digested in microwave digestion system (CEM, Model MARS Xpress) at 120 ^o^C for 10 min according to the EPA method 3015-8 (USEPA, 2007; Sosinski and Sze 1991). After cooling to room temperature, 20 ml of DI water was added to the digested solution with the inner wall and lid thoroughly rinsed and transferred into centrifuge tube where centrifugation was carried out using Eppendorf centrifuge 5702 for 5 minutes at 3000 rpm before filtering through Whatman filter paper No. 42 into 50 ml volumetric flask and diluted with DI water to the mark. Reagent blank was also prepared like the sample but without adding soil solution in triplicates. The total concentrations of heavy metals were determined by Inductive Coupled Plasma Optical Emission Spectrometry (ICP-OES) (Thermo Scientific iCAP 6000 series). The ICP was calibrated with a mixture of standard solutions of the metals of interest.

**Background PAHs determination**

PAHs originate from both natural and anthropogenic sources. The anthropogenic sources include release of petroleum products such as crude oil (petrogenic) (Oluseyi, *et al*., 2011, Kowalewska & Konat, 1997) and from combustion and pyrolysis of fossil fuels or wood (pyrolytic). An extraction of PAHs from soil samples especially the contaminated soil sample to establish a baseline study was achieved ultrasonically using modified methods of Fan *et al.* (2008) and Song *et al.* (2006). Air dried soil (5 g) was weighed and mixed with 25 ml of DCM and extracted for 3 successive times for 1 h sonication using the ultrasonic bath (Clifton sw30H) in which the water temperature was kept at 35 ^o^C in other to optimize the PAHs extraction efficiency. The mixture was centrifuged using Eppendorf centrifuge 4000 rpm for 5 min to separate the supernatant from the soil and filtered into 20 ml vials where it was stored in the refrigerator at 4 ^o^C in preparation for clean-up and analysis. Solid phase extraction (SPE) clean-up was carried out with a 12 port vacuum manifold from SUPELCO with 1 g 6^-1^ ml ENVI^TM^-Florisil glass cartridges. After conditioning the sorbent of the SPE cartridges, 3 ml of the supernatant was filtered through the column and was consecutively eluted with 6 ml hexane and dichloromethane mixture of 1:1. The combined eluate was completely dried under the gentle stream of nitrogen, and then re-constituted in hexane with a final volume of 2 ml for GC- FID analysis. Samples extracts (1 µl) were analysed by a Shimadzu GCMS–QP 2010 and a DB-5 capillary column (30 mm x 0.25 mm x 0.25 µm). Separation was achieved according to the following program: the initial oven temperature was 80 ^o^C (held time for 1 min), and increased to 275 ^o^C at 15 °C min^-1^, held for 1 min: and then to 285 ^o^C at 10 °C min^-1^, held for 1 min: after that increased to 295 ^o^C at 5 °C min^-1^, held for 1 min. Helium was used as the carrier gas (1.5 ml min^-1^) and make up gas (35 ml min^-1^). A 1.0 µl aliquot of the extract was injected in the splitless mode. The injector was held at 250 ^o^C and the detector at 300 ^o^C.
